# A sensitive scoring system for the longitudinal clinical evaluation and prediction of lethal disease outcomes in newborn mice

**DOI:** 10.1038/s41598-019-42414-4

**Published:** 2019-04-11

**Authors:** Beate Fehlhaber, Anna S. Heinemann, Kathrin Rübensam, Maike Willers, Lena Völlger, Sandra Pfeifer, Maren von Köckritz-Blickwede, Dorothee Viemann

**Affiliations:** 1 Department of Paediatric Pneumology, Allergology and Neonatology, Hannover Medical School, 30625 Hannover, Germany; 2 Research Centre for Emerging Infections and Zoonoses (RIZ), University of Veterinary Medicine Hannover, 30559 Hannover, Germany; 3 Department of Physiological Chemistry, University of Veterinary Medicine Hannover, 30559 Hannover, Germany

## Abstract

Neonatal animal models are increasingly employed in order to unravel age-specific disease mechanisms. Appropriate tools objectifying the clinical condition of murine neonates are lacking. In this study, we tested a scoring system specifically designed for newborn mice that relies on clinical observation and examination. Both, in a neonatal sepsis model and an endotoxic shock model, the scoring results strongly correlated with disease-induced death rates. Full as well as observation-restricted scoring, reliably predicted fatality and the remaining time until death. Clinical scores even proved as more sensitive biomarker than 6 traditionally used plasma cytokine levels in detecting sepsis at an early disease stage. In conclusion, we propose a simple scoring system that detects health impairments of newborn mice in a non-invasive longitudinal and highly sensitive manner. Its usage will help to meet animal welfare requirements and might improve the understanding of neonatal disease mechanisms.

## Introduction

Neonatal mouse models are increasingly employed in order to approach to urgent scientific questions related to the neonatal period of life. In particular, septic diseases are still one of the leading causes of death in newborn infants worldwide^1^. To gain better insights into the newborn’s immunity and the pathogenesis of neonatal sepsis, animal models need and needed^2–9^ to be applied in newborn mice, not least because of ethical reasons. Moreover, numerous investigations including mouse experiments have been initiated that study the imprinting effects and long-term consequences of early childhood challenges and events on the development of health^2–9^. However, the longitudinal assessment of laboratory markers is extremely difficult in neonatal mice and appropriate tools are lacking that allow evaluating their clinical condition in an objectified and sensitive manner.

Evaluating the health status of experimental animals can be realized by using invasive and non-invasive methods. According to European guidelines, the highest priority of keeping, breeding and using animals in academic research is the wellbeing and protection of these animals^10^. Therefore, non-invasive methods are preferable to reduce pain and suffering to a minimum^11^. For adult mice, a few scoring systems have been proposed, mostly considering specific clinical symptoms of the modelled disease^12–14^. Appropriate schemes for the objectified clinical evaluation and follow-up of the health status and welfare of neonatal rodents are lacking, while invasive studies, e.g. blood drawings, are extremely difficult to accomplish in a longitudinal manner. Scoring systems for adult animals can not readily be transferred to neonatal animals due to the developmental and anatomic peculiarities of newborn pups like the absence of fur and posture, different movement patterns or the restricted accessibility of eyes. Even loss of weight is often a useless parameter as it increases physiologically in the first weeks of life in an exponential manner. Thus, a clinical assessment tool for newborn mice is urgently needed to comprehensively capture age-specific response patterns to diseases and meet animal welfare requirements.

In this study, we propose a scoring system for newborn mice that is based on observation and minimal physical examination. The value of the scoring system was studied by applying two disease models with different death kinetics in two mouse strains with different disease susceptibilities. Evaluation criteria were the correlation between scoring results and disease outcomes, the predictive accuracy of the scoring in terms of fatality and remaining time until death and its sensitivity in comparison to common invasive laboratory markers.

## Results

### Score sheet for newborn mice

**Figure 1:**
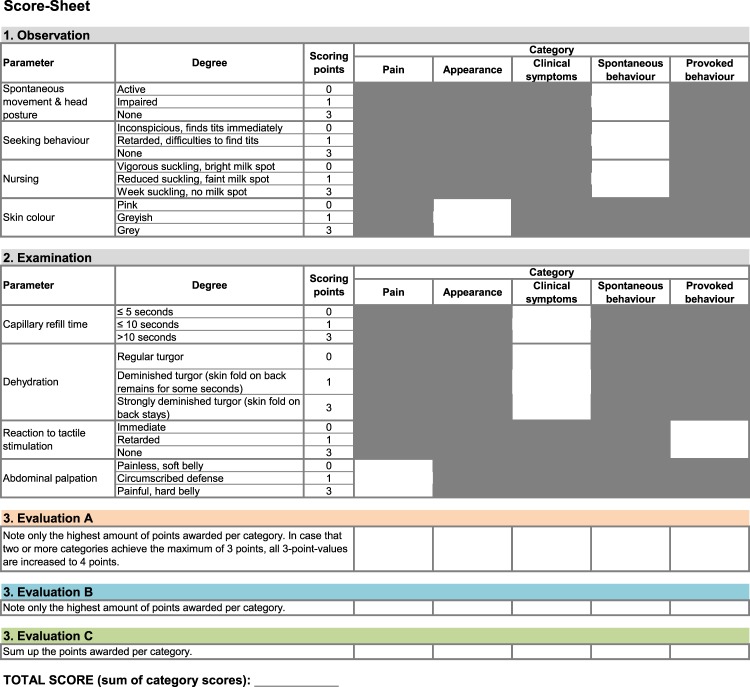
Score sheet. Documentation sheet for scores awarded to individual mice at relevant time points. Scoring was based on observation and examination of indicated parameters to assess the different categorical aspects of the health status. Three different algorithms (evaluation method A, B, C) were used to calculate the total scores per category which subsequently were summed up to a final total score.

**Figure 2:**
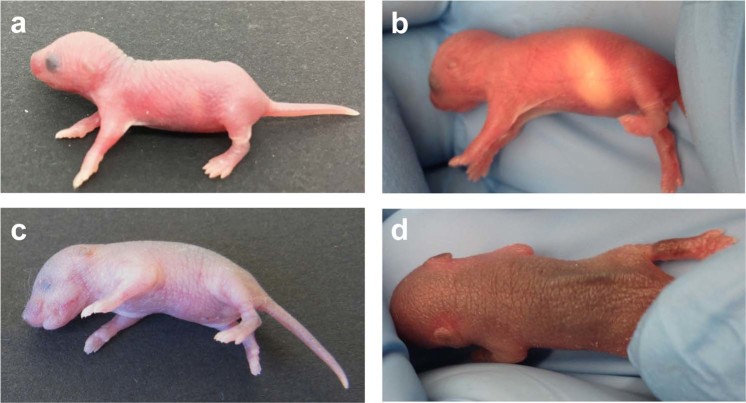
Typical appearance of observational scoring parameters in healthy and diseased newborn mice. (**a**) Healthy newborn pup with active raised head posture, vivid spontaneous movement and rosy-pink skin colour. (**b**) Bright abdominal milk spot indicating normal suckling behaviour and nursing. (**c**) Newborn mouse infected with *S. aureus* presenting with impaired head posture, no spontaneous movement and greyish skin colour. (**d**) Decreased skin turgor in a septic neonate with folds of the back skin remaining elevated due to dehydration.

Due to obvious developmental differences between adult and newborn mice we designed an adapted score system for neonates considering the lack of fur, accessibility of skin colour, reduced movement, nursing/respective seeking behaviour and visibility of abdominal milk spots. The evaluation of newborn animals included observational and examination-based parameters to assess different categorical aspects of the health status, i.e. pain, appearance, clinical symptoms, spontaneous behaviour and provoked behaviour. The parameters were listed in a score sheet (Fig. 1) that was used to record the scores awarded to individual mice at defined time points. The score values of each category were summed up to a final total score, while three different strategies (A, B, C) were tested to calculate the score per category (Fig. 1). In strategy A, only the highest amount of points awarded per category was noted. In case that two or more categories achieved the maximum of 3 points, all 3-point-values were increased to 4 points. Evaluation strategy B required to note only the highest amount of points awarded per category. For evaluation according to strategy C, all points awarded per category were summed up. Additionally, we compared full scoring including observational and examination-based scoring (A_full, B_full, C_full) with the scoring that was restricted to observational parameters (A_obs, B_obs, C_obs). In Fig. 2, healthy and diseased neonates are shown to illustrate exemplarily clear differences in selected observational scoring parameters.

### Robustness of clinical scoring

**Figure 3:**
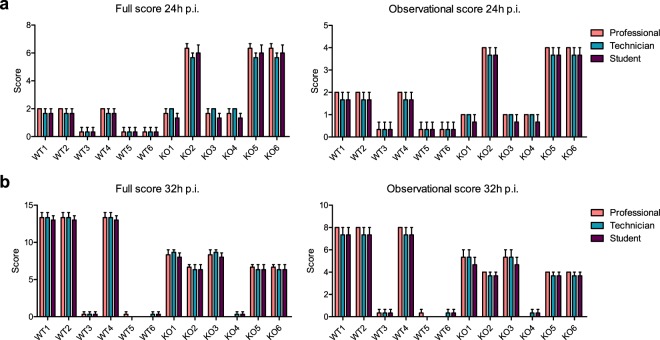
Inter-observer variability of clinical scoring. Newborn mice (d2) (WT *n* = 6, *s100a9*^−/−^ (KO) *n* = 6, each from two litters) were injected subcutaneously with *S. aureus* (7 × 10^4^ CFU) and 24 h (**a**) and 32 h (**b**) later independently scored by three professionals, technicians and students, respectively. Final full scores and observation-restricted scores were computed according to strategy C (summation of scoring points per category). Bars show means ± SD of the scores awarded to the individual mice.

In a first step, we tested the proposed scoring system in newborn C57BL/6 mice (wildtype, WT) and the more vulnerable *s100a9* knock-out (*s100a9*^−/−^) mice^15^ in an established model of *Staphylococcus (S.) aureus*-induced neonatal sepsis^3^ and determined the inter-observer variability of scoring. For that purpose, three blinded experienced scientists, three animal keepers and three medical students who have passed the obligatory animal experiment course at the central animal facility of the Hannover Medical School independently assessed *n* = 12 individual *S. aureus*-challenged newborn mice 24 hours (h) and 32 h post infection (p.i.) (Fig. 3a,b). The variance of awarded scores within each observer group was very low and in none of the mice significant differences were detected between the scoring results of the respective observer groups. The data suggest that the score system is robust and applicable by trained examiners with a limited amount of observer experience. In the following main studies, we used the mean of scores awarded independently by one experienced scientist, one animal keeper and one medical student.

### Clinical scoring correlates with sepsis-induced death rates in newborn mice

**Figure 4:**
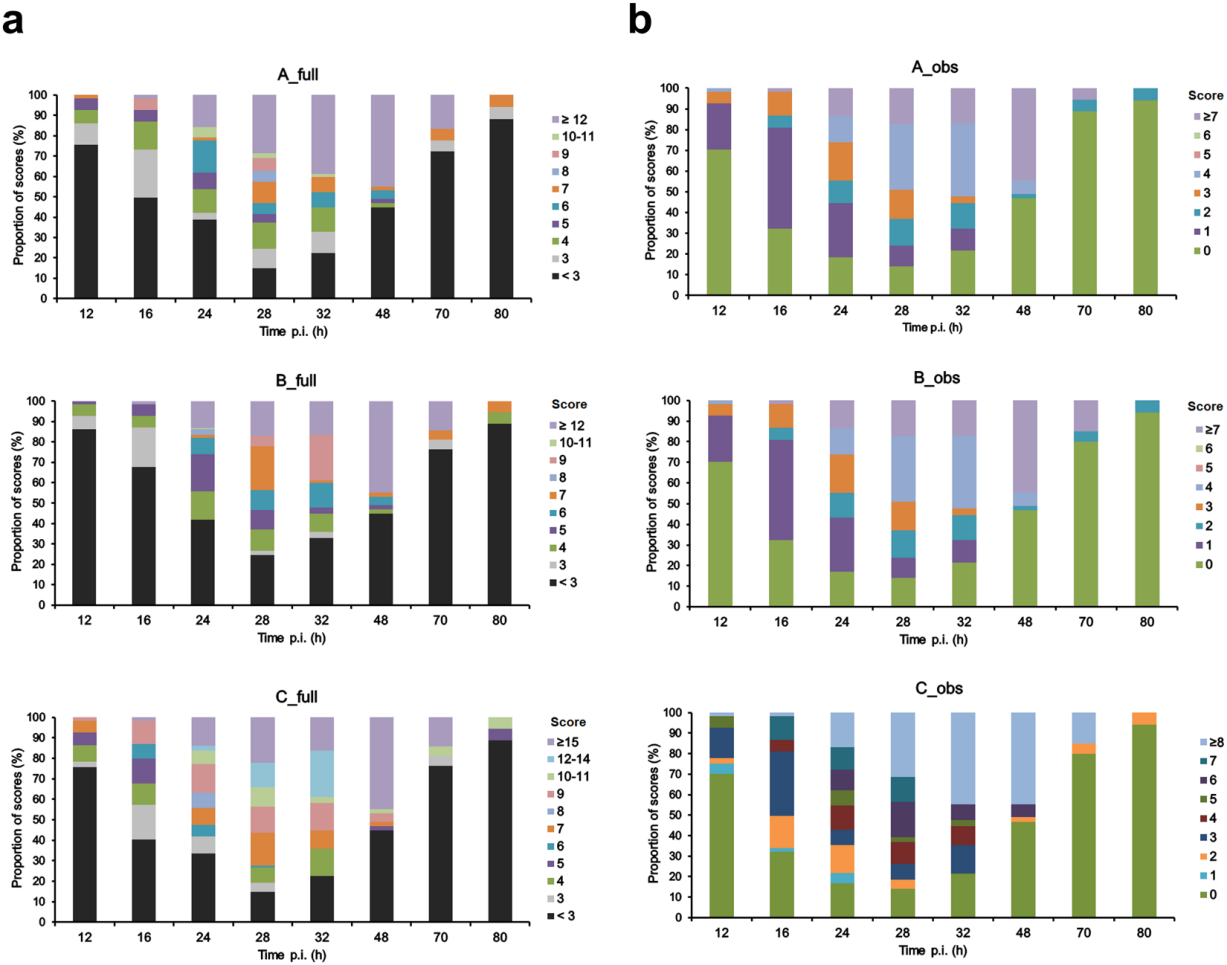
Clinical scoring results over the course of sepsis in newborn mice. (**a,b**) Sepsis was induced in newborn mice (d2) (WT *n* = 50 from 6 litters, *s100a9*^−/−^ *n* = 74 from 11 litters) by subcutaneous injection of *S. aureus* (7 × 10^4^ CFU). Bars show the distribution of awarded full scores (**a**) and scores restricted to observational parameters (**b**) that have been calculated based on the evaluation strategies A, B and C at the respective time points after infection (p.i.). Scores are represented by distinct colours as indicated.

**Figure 5:**
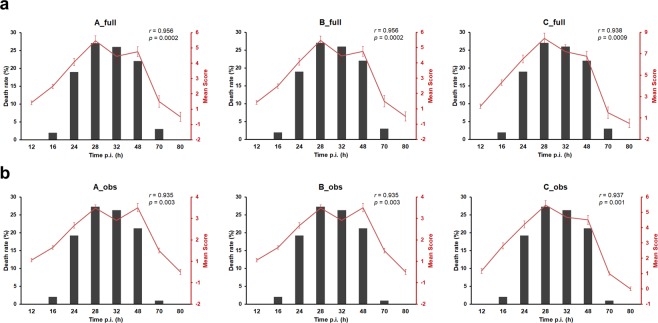
Correlation between clinical scoring and sepsis-induced death rates in newborn mice. (**a,b**) Sepsis was induced in newborn mice (d2) (WT *n* = 50 from 6 litters, *s100a9*^−/−^ *n* = 74 from 11 litters) by subcutaneous injection of *S. aureus* (7 × 10^4^ CFU). Bars show the death rates at indicated time points after infection (p.i.). The graphic red lines indicate means ± SEM of awarded full scores (**a**) and scores restricted to observational parameters (**b**), that have been calculated based on the evaluation strategies A, B and C at the respective time points after infection (p.i.). *r*, Pearson’s correlation coefficient; *p*, *p* value.

Next, we interrogated how the scores are distributed during the longitudinal course of *S. aure*u*s*-induced neonatal sepsis and whether they correlate with the death rates. After challenge with *S. aureus*, 124 newborn mice (50 WT and 74 *s100a9*^−/−^) were observed for 80 h and longitudinally scored at defined times points. After death the respective mouse was excluded from further follow-up scoring. The numbers of scored mice were *n* = 124 at 12 h, *n* = 124 at 16 h, *n* = 122 at 24 h, *n* = 94 at 28 h, *n* = 67 at 32 h, *n* = 49 at 48 h, *n* = 21 at 70 h and *n* = 18 at 80 h. The distribution of awarded scores at the respective scoring time points is shown in Fig. 4. During the first 12 h p.i., most of the mice (75%) showed no significant symptoms (scores <3). Subsequently, the proportion of scores >3 slowly increased suggesting beginning of sepsis. From 28 h p.i. until 48 h p.i, 50% of the mice had full score values of ≥6 with evaluation method A_full and B_full (30% respective 40% of the maximal achievable score), and ≥9 with C_full (38% of the maximal achievable score) (Fig. 4a), reflecting the overt impairment of their health status, respectively. The analogue values for the observation-restricted evaluation were ≥3 with A_obs and B_obs (38% respective 50% of the maximal achievable score), and ≥6 with C_obs (50% of the maximal achievable score) (Fig. 4b). Thereafter, scores of surviving mice decreased again indicating recovery from sepsis. The mean final scores clearly correlated with the kinetics of death rates at the time points of evaluation with the full scoring (A_full and B_full) performing slightly better (Fig. 5a) than the scoring restricted to observational parameters (Fig. 5b). These findings demonstrated that the scoring system is a reliable method to measure and to record the severity of the clinical course of sepsis in newborn mice.

### The scoring system predicts fatal courses of sepsis in newborn mice

**Figure 6:**
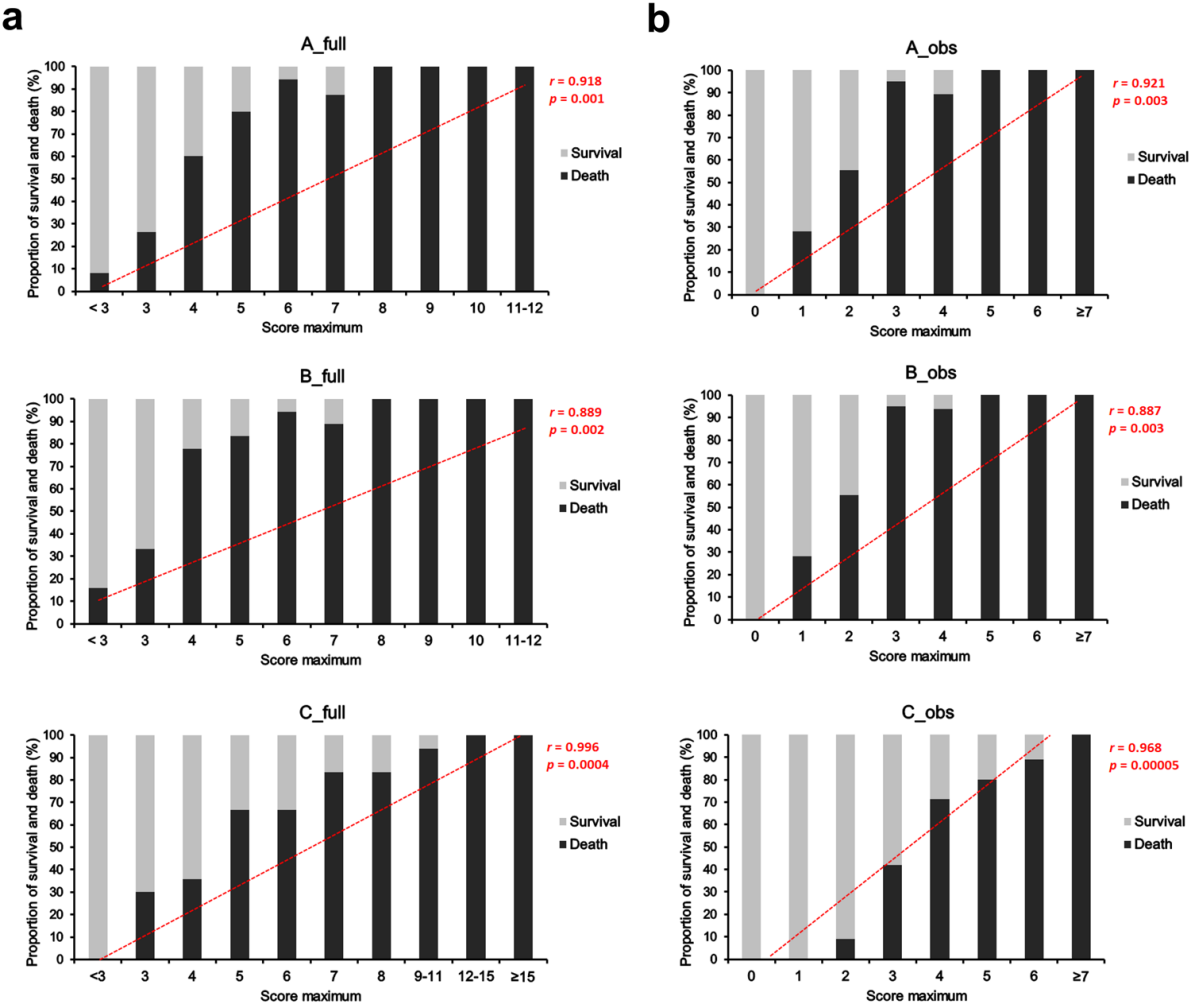
Clinical scoring predicts sepsis fatality in newborn mice. After sepsis induction, the newborn mice (WT *n* = 50 from 6 litters, *s100a9*^−/−^ *n* = 74 from 11 litters) were assigned to score maximum groups according to the highest score value they were awarded during the 80 h of evaluation upon full scoring (**a**) and sole observational scoring (**b**) computed with strategy A, B or C. Bars represent the proportion of survival and death within the score maximum groups. Dotted red lines indicate the slope of the linear correlation between the proportion of death and the highest score awarded before death. *r*, Pearson’s correlation coefficient; *p*, *p* value.

**Figure 7:**
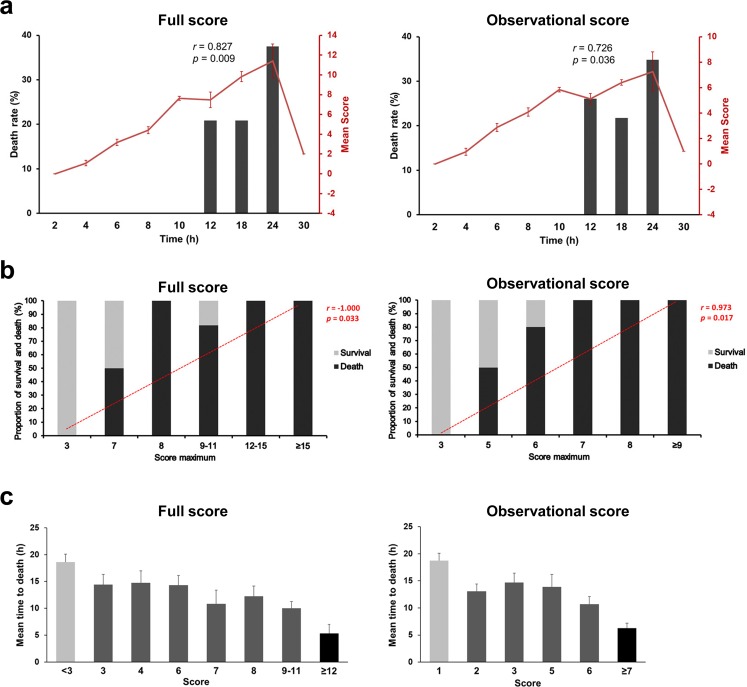
Predicting the time until death by clinical scoring. For each final full score and observation-restricted score awarded during the course of *S. aureus* infection the mean time until death from sepsis/respective end of evaluation time was determined (*n* = 50 WT neonates from 6 litters and *n* = 74 *s100a9*^−/−^ neonates from 11 litters). Results are plotted as bars + SEM. Colours summarize 3 different categories of time to death: light grey ≥24 h, dark grey 16 h to 23 h, black ≤12 h.

To assess the accuracy of the scoring system to predict fatal courses of sepsis we determined the highest score value each animal had been awarded during the evaluation period of 80 h and assigned the animals to respective score maximum groups. Subsequently, the final outcome of the animals within these groups was determined and plotted as proportion of death and survival (Fig. 6). The occurrence of death and the highest score value awarded prior to death correlated best when final scores were calculated according to strategy C with the full scoring (Fig. 6a) correlating slightly better than the observation-restricted scoring (Fig. 6b). Here, a once awarded full score of ≥5 respective an observation-restricted score of ≥4 indicated that about 65–70% of such scored mice would die, while a full score of ≥12 respective an observation-restricted score of ≥7 predicted definitive fatality (100%). Thereby, the mean remaining time until death occurred was 4 h at a full score of ≥12 and, somewhat less exact, 7 h at an observational score of ≥7 (Fig. 7). These findings demonstrated that the proposed clinical scoring system is a sensitive indicator of fatal courses of sepsis, allowing early termination of an experiment to avoid unnecessary suffering of newborn experimental mice.

### The scoring system for newborn mice is suitable for the evaluation of diseases with different clinical kinetics

**Figure 8:**
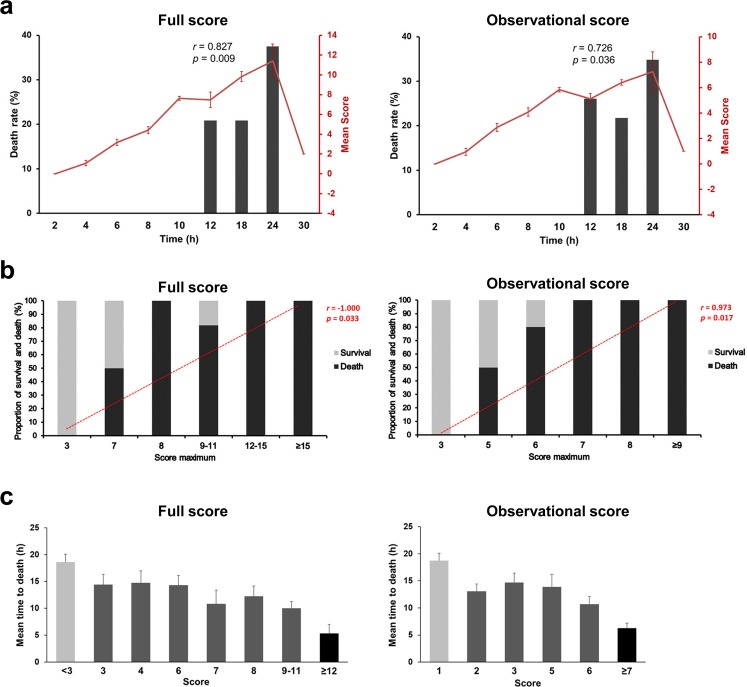
Clinical scoring in a neonatal endotoxic shock model. (**a**–**c**) Endotoxemia was induced in newborn mice (d2) (WT *n* = 15 from 2 litters, *s100a9*^−/−^ *n* = 9 from 2 litters) by intraperitoneal injection of 20 µg LPS. Final full scores and scores restricted to observational parameters were computed according to evaluation strategy C. (**a**) Death rates are plotted as bars. The graphic red lines indicate the means ± SEM of awarded scores at indicated time points after LPS challenge. (**b**) The proportions of survival and death within score maximum groups are shown as bars. Score maximum groups were assigned based on the highest score value awarded to a mouse during the 30 h of evaluation after LPS challenge. Dotted red lines indicate the best fit of the linear correlation between the proportion of death and the highest score awarded prior to death. *r*, Pearson’s correlation coefficient; *p*, *p* value. (**c**) The time until death respective end of observation time of indicated scores is plotted as mean + SEM. Colours summarize 3 different categories of time to death: light grey ≥16 h, dark grey 7 h to 14 h, black ≤6 h.

To verify whether the proposed scoring system is also appropriate for the evaluation of diseases with clinical courses that differ from that of bacterial sepsis we applied an established neonatal endotoxic shock model^2,16^ to 24 newborn mice. Full as well as sole observational scoring was performed at defined time points during an evaluation period of 30 h. The numbers of scored mice were *n* = 24 at 2 h, *n* = 24 at 4 h, *n* = 24 at 6 h, *n* = 24 at 8 h, *n* = 24 at 10 h, *n* = 21 at 12 h, *n* = 16 at 18 h, *n* = 11 at 24 h and *n* = 3 at 30 h. The final scores were computed by summation of all scoring points per category, respectively (strategy C). As shown in Fig. 8a, this model was characterized by a much faster kinetic compared to the sepsis model (Fig. 5). Deaths of newborn mice occurred already after 10 h and peaked between 20 h and 24 h after LPS challenge, while survival beyond 24 h meant complete recovery. The mean final scores at the defined monitoring time points correlated well with the course of death rates; similar as in the sepsis model full scoring performed slightly better than scoring restricted to observational parameters (Fig. 8a). Furthermore, the occurrence of death correlated excellently with the highest score value awarded before death with full and observational scores being comparable (Fig. 8b). Like in the sepsis model, a full score of ≥12 respective an observation-restricted score of ≥7 predicted definitive fatality. The mean remaining time until death in this disease model was 5 h at a full score of ≥12 and, again somewhat less exact, 6 h at an observational score of ≥7 (Fig. 8c). Taken together, these results showed that the here proposed scoring system reliably records the clinical state of newborn mice, regardless of the kinetics of the induced disease.

### Clinical scoring outperforms plasma cytokine levels in monitoring the disease state of septic newborn mice

**Figure 9:**
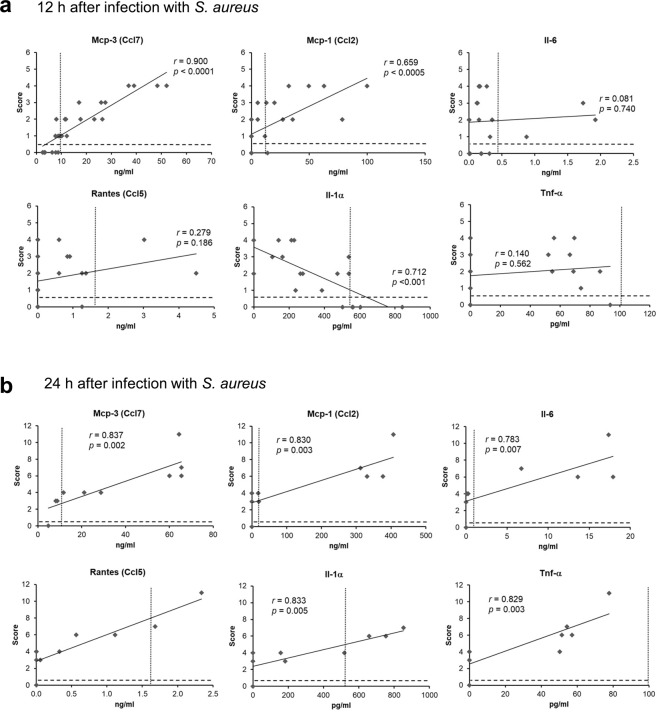
Clinical scoring is more sensitive than plasma cytokine levels to monitor early disease states in septic newborn mice. Plasma cytokine levels of *S. aureus*-challenged neonates (d2) were correlated with the scoring results in these mice as determined directly before killing at (**a**) 12 h (*n* = 14 WT and *n* = 12 *s100a9*^−/−^) and (**b**) 24 h (*n* = 5 WT and *n* = 5 *s100a9*^−/−^) after infection. Scatter plots indicate the best fits, the coefficients *r* and the *p* values of correlation. Lines indicate the cut-offs (mean + 2 SD) of scores (dashed lines) and cytokine levels (dotted lines) in the group of PBS-injected control neonates.

In humans as well as in animal experiments, cytokine and chemokine levels in the plasma are used as biomarker of sepsis and the success of treatment^17–19^. To corroborate the value of the proposed non-invasive scoring system we determined the plasma levels of Ccl7 (alias monocyte chemoattractant protein 3, Mcp-3), Ccl2 (alias Mcp-1), Il-6, Ccl5 (alias regulated on activation, normal T cell expressed and secreted, Rantes), Il-1α, and Tnf-α in 36 *S. aureus*-infected newborn mice 12 h p.i. (Fig. 9a) and 24 h p.i. (Fig. 9b). Cytokine levels were correlated with the clinical score (C_full) awarded at these time points. Already 12 h p.i., the scores of *S. aureus*-infected mice were higher (between 1 and 4) than the scores of PBS-treated control mice, reflecting the beginning of sepsis. At this early stage, only the plasma levels of Ccl7 correlated well and those of Ccl2 mediocrely with the scoring results (Fig. 9a), while the plasma levels of Il-6, Ccl5, Il-1α and Tnf-α of infected mice did not correlate with the clinical scoring and were not increased compared to PBS-treated control mice (Fig. 9a). Only after 24 h of infection, the plasma levels of all cytokines correlated with the scoring results (Fig. 9b). However, at 24 h p.i., the clinical scoring still discriminated better between non-infected and *S. aureus*-infected mice than the levels of Ccl5, Il-1α and Tnf-α. The latter were in most of the infected animals not higher than in control mice suggesting insufficient specificity of the cytokine assay at least with respect to these cytokines to discriminate infection-induced increases in neonates (Fig. 9b). Collectively, these data demonstrated that the proposed clinical scoring highly sensitively indicates beginning sepsis in a newborn mouse, therein outmatching the value of common plasma cytokine levels as early biomarker. The correlation between scores and plasma cytokine levels at later stages of sepsis corroborates the quality of the scoring system as an objective follow-up parameter.

## Discussion

Clinical scoring systems are widely used in animal research to evaluate the health status of experimental animals as an objective scientific parameter and for evaluating the animal welfare. In mice, validated scoring systems are only published for adults^11–13^ but are missing in neonates. In order to assess important scientific questions relating to early life immunity like the pathogenesis of neonatal sepsis and the imprinting and maturation of immunity^2–9^, a clinical scoring system is needed that allows the reliable non-invasive evaluation of newborn experimental animals in a longitudinal manner. A previously proposed score sheet for the welfare assessment of neonatal transgenic mice included only two parameters, namely whether pups are in or out of nest and presence of milk spot^20^. The accuracy of this scoring system has never been validated. In our hands, considering only these scoring parameters was insufficient to determine the clinical condition of a newborn mouse. Pups out of nest indicates already an end stage of disease. In turn, most pups at a high morbidity stage are still in the nest. Likewise, the milk spot alone is not suited since its presence depends on the time of last suckling of the individual mouse and is often missed even in completely healthy murine neonates. Very recently, Brook *et al*. proposed a monitoring strategy for sepsis-diseased 7-day-old mice that uses the ability to right themselves and the level of hip mobility to define health scores^21^. Although this scoring system is easy to perform, these parameters are not applicable in newborn mice below 7 days because at this age the mobility is naturally restricted to crawling and the capability to right is not yet developed.

In this study, we proposed and tested a scoring system for newborn mice that closely followed clinical evaluation parameters used in human neonates. The clinical signs in diseased human neonates are usually unspecific and often subtle. The APGAR score is the most established score system in human neonates and includes appearance, heart rate (pulse), responding to tactile stimuli (grimace), activity and respiration^22^. A more sepsis specific scoring system for human neonates is the Neonatal Therapeutic Intervention Scoring System (NTISS) that considers 62 parameters addressing to respiratory, cardiovascular and metabolic/nutritional impairments, extent of drug therapy and transfusions, need of monitoring and invasive procedures, and type and number of vascular accesses^23^. Some characteristics of newborn mice are of advantage; the absence of fur allows evaluating parameters inaccessible in adult mice, e.g. skin colouring or presence of milk spot as an indirect criterion for the suckling behaviour. Based on these considerations, we designed a scoring system for newborn mice that included observation and minimal physical examination and avoided separation of the pups from the mother. The application of the proposed scoring system on newborn mice after infection with *S. aureus* or induction of endotoxin-mediated inflammatory shock yielded highly significant correlations between the health impairment/respective death rates and score values. In both models, clinical scoring was able to predict mortality and allowed conclusions on the remaining time until death that was dependent on the kinetic and lethality of the respective model. The comparison of three different strategies for the computation of the final score revealed that scores best achieved prediction of mortality if all scoring points awarded for each parameter had been summed up (strategy C), equally applying for full and observation-restricted scoring.

Physical examination was used to assess pain, signs of circulatory failure and the behaviour upon tactile stimulation. Since examining experimental mice is time consuming, eventually difficult, and most importantly, might be noxious to the mice due to thereby imposed stress, we checked whether the clinical scoring could be restricted to observational parameters. Surprisingly, despite a slightly less accurate predictability of the time until death, our results revealed that assessing the appearance and the spontaneous behaviour of murine neonates allowed a sufficiently reliable evaluation of the health status and imminent deaths in neonatal mice. Consequently, sole observational scoring can be recommended as tool to assess the welfare of diseased newborn experimental mice.

Finally, clinical scoring proved as a more sensitive biomarker of beginning sepsis in neonates than plasma cytokine levels. The reasons might be both the known inflammatory hyporesponsiveness of newborn mice^2,3,16,24^ together with an insufficiency of currently available cytokine assays to reliably detect cytokine levels at low ranges. These findings were in line with the well-known dilemma in human neonates that cytokine levels insufficiently indicate imminent septic events while clinical evaluation is still the most sensitive method^14,25,26^. Of note, Il-6, the most common sepsis biomarker used in human neonates, was only at the late stage of sepsis (24 h p.i.) increased in newborn mice.

In order to ensure wellbeing and protection of experimental animals individual monitoring schedules have to be established depending on the kinetics of the experimental disease model and the susceptibility of the used mouse strain. We suggest the usage of our clinical scoring system to determine when first disease signs and deaths occur in other neonatal disease model than *S. aureus*-mediates sepsis or endotoxic shock. Subsequently, such scoring-based data might be used to establish disease model-specific and mouse strain-adapted monitoring schedules focussing on those phases of illness when deaths start and peak until survival is ensured.

In summary, the here proposed scoring system proved as the first sensitive tool that provides reliable longitudinal information on the health status of a newborn mouse and was ahead of common invasive laboratory markers in indicating the beginning of illness in case of sepsis. Clinical scoring allowed predicting lethal outcomes of murine neonates at an early disease stage which might aid in meeting animal welfare requirements.

## Materials and Methods

### Mice

C57BL/6 WT mice (Charles River, Sulzfeld, Germany) and *s100a9*^−/−^ mice^15^ were used for breeding and housed under specific pathogen-free conditions at the Central Animal Facilities at Hannover Medical School and the University of Veterinary Medicine Hannover and maintained under standard conditions according to institutional guidelines. Both mouse strains were constantly bred, and litters were used randomly. For experiments, neonates were used at the age of 2 days (d2) and only if the mothers had already given birth three times at minimum. No animals needed to be excluded from the studies.

### Ethical approval

Mouse experiments were carried out in accordance with German Animal Welfare Legislation and performed as approved by the Lower Saxony State Office for Consumer and Food Safety, Germany (approval no. 33.12-42502-04-14/1682, 33.12-42502-04-15/1951 and 33.12-42502-04-15/1969).

### Scoring of newborn mice

Animals were observed for at least 5 min to assess spontaneous movement and head posture, seeking behaviour, nursing and skin colour. Parameters that required physical examination were the capillary refill time to evaluate the circulatory status, the skin turgor to detect signs of dehydration, tactile stimulation to test flight reflexes and alertness, and abdominal palpation to assess unconscious defence reactions due to pain. Appraisal of the milk spot shining from the stomach through the abdomen^27^ was also considered when analysing the nursing behaviour. For each parameter scoring points were awarded for deficiencies of the health status, rated in terms of no (0 point), moderate (1 point) or significant (3 points) impairment.

### Neonatal disease models

Newborn d2 WT and *s100a9*^−/−^ mice were carefully lifted from their nest and placed onto soft papers on a Styrofoam underground. Forceps with rubber tips were used to hold the animal tight for treatment. Bacterial sepsis was induced by subcutaneous injection of 20 µl of bacterial suspensions containing 7 × 10^4^ CFU *S. aureus* strain Newman (GenBank accession number AP009351.1) in the back of the neonates. Endotoxic shock was induced by intraperitoneal injection of 20 µg lipopolysaccharide (LPS) from *Escherichia coli* 055:B5 (Sigma, Steinheim, Germany) in 20 µl PBS. Mice injected with 20 µl PBS served as controls, respectively. The procedures were performed calm and uninterrupted within minutes to ensure reacceptance of the neonate by the mother. Mice were monitored for survival over a time period of 80 h in the sepsis model and 30 h in the endotoxic shock model. For cytokine studies in the sepsis model, mice were sacrificed by decapitation 12 h and 24 h after bacterial inoculation to harvest blood.

### Cytokine assays

Blood was collected using heparinized glass capillaries and transferred into heparinized tubes. After centrifugation at 500 × g for 5 min, plasma was removed and centrifuged at 2000 × g for 5 min and stored at −80 °C until cytokine analysis was performed using the LEGENDplex Mouse Multi-Analyte Flow from BioLegend (San Diego, USA) according to manufactures’ instructions. Samples were analysed with a FACS Canto II flow cytometer (BD Biosciences, Heidelberg, Germany). Data were processed using DIVA software v8.0.1 (BD Biosciences) and LEGENDplex Data Analysis Software v7.0 (BioLegend).

### Statistics

For correlation analyses, linear regression was computed, Pearson’s correlation coefficient *r* determined and trend lines (best fits) indicated.

## Data Availability

The authors declare that all data supporting the findings of this study are available within the article. Additional data are available from the corresponding author upon reasonable request.
